# Laboratory medicine in pandemic of COVID-19

**DOI:** 10.11613/BM.2022.020501

**Published:** 2022-04-15

**Authors:** Leida Tandara, Petra Filipi, Daniela Supe Domic, Branka Kresic, Ivo Ivcic, Sanda Stojanovic Stipic, Zana Rubic, Marijan Tandara

**Affiliations:** 1 Department of Medical Laboratory Diagnostic, University Hospital Split, Split, Croatia; 2 University of Split School of Medicine, Split, Croatia; 3 University Department of Health Studies, University of Split, Split, Croatia; 4 Clinic for Infectious Diseases, University Hospital Split, Split, Croatia; 5 Department of Anaesthesiology and Intensive Care, University Hospital Split, Split, Croatia; 6 Department of Clinical Microbiology, University Hospital Split, Split, Croatia; 7 Polyclinic Sparac, Split, Croatia

**Keywords:** COVID-19, GDF-15, infertility, interleukin-6, Guillain-Barré syndrome

## Abstract

After the outbreak in China in the year 2019, severe acute respiratory syndrome Coronavirus 2 (SARS-CoV-2) quickly spread around the world causing a protracted pandemic. Approximately one-third of infections appear to be asymptomatic. Symptomatic disease is characterized primarily by symptoms of respiratory tract infection of varying severity. But Coronavirus Disease 2019 (COVID-19) is much more than an acute respiratory disease because SARS-CoV-2 affects many organs inducing a vast number of symptoms such as cardiovascular, neurological, gastrointestinal, dermatological, with numerous complications. Short and long-term effects of infection, severe ones, and especially mild forms of the disease which affect a huge number of patients need to be further investigated. Laboratory medicine has a crucial role in early diagnosis of the disease, recognition of the patients who need hospital care, and close monitoring of hospitalized patients to timely identify associated clinical complications as well as follow-up of patients with long-term COVID-19.

## Introduction

Since the beginning of 2020, the Coronavirus Disease 2019 (COVID-19) has greatly influenced our personal and professional lives. The pandemic affected all medical specialties, including laboratory medicine. Medical laboratories are necessary for early diagnosis of the disease, monitoring of hospitalized patients, and are involved in epidemiologic surveillance (*1*, *2*). The pandemic has changed the organization of medical laboratories, so the topic of the 32nd annual symposium organized under the auspices of the Croatian Society of Medical Biochemistry and Laboratory Medicine (CSMBLM) imposed itself. This paper gives an overview of the lectures presented at the 32nd annual symposium named ˝Laboratory medicine in the pandemic of COVID-19˝.

### Coronavirus Disease 2019 is much more than acute respiratory disease

After the outbreak in China in the year 2019, COVID-19 quickly spread around the world causing a protracted pandemic. The causative agent, severe acute respiratory syndrome Coronavirus 2 (SARS-CoV-2) is of animal origin but has successfully adapted to the human host. It is transmitted from person to person primarily by the respiratory route *via* droplets and aerosol. Moreover, SARS-CoV-2 is an enveloped RNA virus that on its surface expresses glycoprotein spike (S-protein) which plays a critical role in viral entry into the host cell. Human angiotensin-converting enzyme 2 (ACE2) is a membrane receptor to which S-protein binds enabling entry of the virus into the host cell. Angiotensin-converting enzyme 2 is expressed on the cells of the lungs, heart, kidney, and intestine (*3*). In addition, ACE2 is also present on vascular endothelial cells allowing the virus to attack almost any organ system, including the central nervous system (*4*).

A more severe COVID-19 is seen in the patients who develop an excessive inflammatory response, a so-called cytokine storm, with consequent development of severe pneumonia, acute respiratory distress syndrome (ARDS), or even sudden death. Surprisingly, this type of inflammatory response is frequently found in patients who produce neutralizing antibodies early. A proposed underlying mechanism is an antibody-dependent enhancement (ADE) in which host cells extensively uptake virus-antibody complexes resulting in their enhanced viral infection and massive death with excessive production of proinflammatory cytokines and chemokines (*3*). From a clinical point of view, the most important risk factors for severe COVID-19 are older age, obesity, chronic obstructive pulmonary disease, and other lung diseases, cardiovascular disease, diabetes mellitus, chronic kidney disease, malignancies (in particular haematological malignancies), immune diseases, and iatrogenic immunosuppression following solid organ or hematopoietic stem cell transplantation. Laboratory features that are associated with worse outcomes include lymphopenia and thrombocytopenia, elevated values of liver enzymes, lactate dehydrogenase (LD), inflammatory markers such as C-reactive protein (CRP) and ferritin, inflammatory cytokines such as interleukin-6 (IL-6) and, tumour necrosis factor α (TNF-α), troponin, creatine kinase (CK), D-dimers, prolonged prothrombin time (PT), and acute kidney injury (*5*).

Up to 30% of patients with severe COVID-19 experience complications such as pulmonary embolism, deep vein thrombosis, microvascular thrombosis, as well as arterial embolism with consequent extremity ischemia, cerebral stroke, or myocardial infarction (*6*). These events are the result of hypercoagulability that has two main pathways: hyperinflammation and specific virus-induced disturbance of the renin-angiotensin system. Due to viral uptake of the ACE2 receptor, the availability of activated ACE2 is reduced with a consequent increase in angiotensin II that favours the systemic procoagulant state. In addition, the virus appears to directly increase the concentration of plasminogen activator inhibitor 1 (PAI-1) contributing to the procoagulant state (*7*, *8*).

Approximately one-third of infections appear to be asymptomatic. Symptomatic disease is characterized primarily by symptoms of respiratory tract infection of varying severity: mild, severe, or critical. Almost 80% of patients have mild disease with mild pneumonia or without it. Severe disease with dyspnoea, hypoxia, or > 50% lung involvement was reported in 14%. A critical disease characterized by respiratory failure, shock, or multiorgan dysfunction was reported in 5% of symptomatic patients (*5*). The main disease complications are respiratory failure, cardiovascular events (arrhythmias, myocardial injury, heart failure, and shock), thromboembolism, and neurological complications (most often encephalopathy, and more rarely strokes, seizures, movement disorders, ataxia, motor, and sensory deficits, and Guillain-Barré syndrome (GBS)) (*5*-*16*).

In children, SARS-CoV-2 generally causes mild illness, but, although rarely, it may induce a severe disease named multisystem inflammatory syndrome in children (MIS-C) (*17*-*19*). After an acute illness, a significant number of patients have persistent difficulties such as fatigue, dyspnoea, cognitive and psychological problems (fear, depression, posttraumatic stress disorder) (*20*, *21*).

Detection of viral RNA in nasopharyngeal swabs by reverse transcription-polymerase chain reaction (RT-PCR) is the main diagnostic tool in COVID-19. Currently, no effective antiviral drug is available. Remdesivir was not shown to reduce COVID-19 mortality but may shorten the recovery time of the subgroup of patients that require low-flow oxygen therapy (< 10 L/min). Treatment of severe COVID-19 includes respiratory support, anti-inflammatory therapy (corticosteroids, tocilizumab, baricitinib), and the prevention and treatment of thromboembolic complications with low molecular weight heparin (LMWH).

### “Point-of-care” tests for diagnosis of SARS-CoV-2 infection

Point-of-care (POC) testing is a form of testing in which the analysis is performed at the site of a patient with the result leading to a possible change in the care of the patient (*22*). The gold standard for identification of SARS-CoV-2 is the RT-PCR assay, but it requires trained laboratory staff, expensive equipment, and a long turnaround time (*23*). On contrary, POC tests are easy to perform, using minimal equipment with no complicated preparation steps, and provide results usually within two hours from sample collection (*24*). Nasopharyngeal samples are considered to be adequate for testing both COVID-19 RT-PCR and antigen tests since current research doesn’t provide sufficient evidence for the use of saliva (*25*). The role of POC tests during the COVID-19 pandemic is in the rapid identification of infected people, which then may be followed by quick decisions on their treatment as well as other measures such are isolation and monitoring of contacts.

One of the questions regarding commercially produced POC tests is their accuracy compared with the gold standard. Regarding antigen and molecular tests, World Health Organization (WHO) standards consider different limits of detection, sensitivities, and specificities as acceptable, depending of are the POC tests used for confirmation of suspected SARS-CoV-2 infections in the areas and conditions where routine reference testing is not available or is time-consuming or are they used for both individual and mass testing, for acute and subacute infections as well (*26*).

The acceptable limit of detection for the first mentioned tests, intended for mass public health needs, is equivalent to 10^6^ genomic copies/mL since some studies show an inability to culture virus < 10^6^, which means that such POC test should be accurate enough to detect the most infectious patients (*27*). For the latter mentioned tests, suitable for both individual or mass testing of acute and subacute infections, the acceptable limit of detection is equivalent to 10^3^ genomic copies/mL in any respiratory tract specimen (*26*).

Further, according to WHO standards acceptable sensitivity and specificity for tests used for mass needs are ≥ 80% and ≥ 97% respectively, while for the latter tests used for individual and mass detection are ≥ 95% and ≥ 99% respectively. Noteworthy is that during use in high need circumstances while the prevalence of COVID-19 is low, the positive predictive value (PPV) of the first mentioned tests is < 50% and requires a second confirmation test, while there is no impact on negative predictive value (NPV). In the circumstances of an increased prevalence (10-20%) PPV rises (> 78-89%), while NPV remains in high percentages (95-98%) (*26*).

According to Cochrane systematic review published on March 2021, investigated antigen tests have been shown to be accurate in 72% of symptomatic people who were definitively diagnosed with COVID-19, while in same but asymptomatic ones they were accurate in only 58% (*28*). In not infected people, antigen tests were negative in 96% of symptomatic and 99% of asymptomatic individuals. The accuracy of the test varied considerably between different manufacturers (*28*). Regarding investigated molecular POC tests, they successfully confirmed or ruled out SARS-CoV-2 infection in 95% and 99% of cases respectively (*28*).

According to this Cochrane systematic review results, POC antigen and molecular tests could be used for the diagnosis of SARS-CoV-2 infection in symptomatic individuals if the tests are sufficiently accurate. Antigen tests that meet appropriate WHO criteria could be considered as a replacement for RT-PCR. In the case of low prevalence, positive results require confirmatory testing, while in the case of 20% or higher prevalence, negative results may be considered for verification. More studies of individual tests, as well as test strategies, are needed for making more precise guidelines for POC testing of SARS-CoV-2 infection in cases of mass screening of asymptomatic individuals without known exposure or testing of risk-exposed asymptomatic individuals (*28*).

### Does COVID-19 cause changes in blood cell morphology?

Although technological advances in automated haematology analysers reduced the number of samples that require microscopic blood smear review it remains an important and invaluable tool in patient management. Various diseases change blood cell morphology and microscopic blood smear examination is a simple and often the first test that can direct the clinician to sometimes definitive and more often differential diagnosis as well as prognosis.

Quantitative haematological abnormalities in COVID-19 and their prognostic importance have been well described in the literature and less attention was given to blood cell morphology. Although data on this topic is limited and studies are done on a small number of patients, all showed that COVID-19 infection causes some changes in blood cells morphology (*29*). The most common findings were the presence of large granular, lymphoplasmoid, and Downey cell-like reactive lymphocytes, pseudo Pelger-Huet anomaly, and morphologically changed monocytes (*30*-*40*). Other changes less frequently reported were smudged and apoptotic neutrophils and neutrophils with other dysplastic like changes like C shaped, ring-shaped, and fetus-like nuclei and nucleoplasmic elongations (*30*, *31*, *36*, *37*). Lymphocyte changes did not correlate with patients’ clinical course, but it has been suggested that COVID-19 could become a new aetiology for reactive lymphocytes and can maybe indicate the presence of SARS-CoV-2 before PCR confirmation (*31*-*33*, *37*).

Infection with COVID-19 unquestionably changes blood cell morphology and these along with quantitative abnormalities are important findings in terms of research on hyperinflammation and SARS-CoV-2 effect on blood cells, but the common conclusion is that reliability and importance of these findings in patient prognosis and management is still not clear and needs to be confirmed on lager studies. Also, only a few studies compared morphology findings to COVID-19 negative patients with similar symptoms, blood cell count, or acute respiratory failure (*37*, *39*, *40*). Data about anti-inflammatory therapy was also mostly not presented.

From a haematology laboratory point of view, there are a few things regarding described changes that can be rather confusing. One is the nomenclature, especially in describing monocyte and lymphocyte morphology. Monocyte changes that usually present the same cells were described as either atypical, activated, or vacuolized, and lymphocyte as a variant, reactive or atypical. The others are neutrophil dysplastic-like changes (bizarre nucleus) feature of myelodysplastic syndrome, and some non-clonal disorders (*41*). Counting neutrophiles with bizarre nucleus during routine blood smear examination of COVID-19 patients would probably be very subjective and also question is how to report them.

To our experience, large percentage of our COVID-19 patients results did not meet our established criteria for blood smear examination, and the most common reasons for microscopic examination were the presence of immature granulocyte flag or thrombocytopaenia.

Microscopic blood smear review, although a simple and important tool, is time-consuming and delays results reporting, and for this reason from the laboratory point of view, it is very important to establish the importance and added value of reviewing blood cell morphology to COVID-19 patient management.

### Disturbances in iron metabolism in SARS-CoV-2 infection: Nutritional immunity or something more

Iron is an essential element for virtually all forms of life. Because both, the host and pathogen, require iron, one of the first responses of our organism to infection is withholding of nutrients in a process termed nutritional immunity (*42*). The most significant part of nutritional immunity is the sequestration of iron mediated by the liver hormone hepcidin (*43*).

Viruses need an iron-replete host to efficiently replicate and cause disease. Some viruses use transferrin receptor 1 (TfR1) to enter cells while others modulate cellular iron metabolism of infected hosts (*44*). Changes in iron homeostasis as a response to viral infection are a pathogen-specific phenomenon and varies with the tropism of the infectious agent and the host hepcidin response (*45*).

An increasing number of papers have been published that demonstrate changes in iron homeostasis in COVID-19 patients, but it is not clear yet if these changes are only part of nutritional immunity or possibly play a role in the pathogenesis of the disease. If the latter is true, iron chelation emerges as reasonable adjuvant therapy for COVID-19 (*46*-*48*).

One of the most important regulators of iron homeostasis during infection is hepcidin. This peptide hormone blocks the absorption and recycling of iron thus modifying intracellular and extracellular iron concentration (*49*). While inflammation and iron overload increase hepcidin expression, hypoxia/anaemia and increased erythropoietic activity decrease it. In COVID-19 hepcidin regulation could be particularly complex. Namely, inflammation and hypoxia simultaneously generate opposing signals on hepcidin expression. Moreover, the strength of these signals is quite different in mild, moderate, and severe diseases dictating net effect on iron homeostasis.

A study by Zhou *et al.* showed increased values of serum hepcidin in severe and mild groups of COVID-19 patients compared to the healthy group. Authors propose that hepcidin and serum ferritin concentrations are possible indicators of COVID-19 severity (*50*). Hepcidin concentrations at admission also predict COVID-19 severity and mortality in a study by Nai *et al.* (*51*). In another study, opposite to these results, decreased hepcidin concentration was found in the critically ill group compared with the control group (*52*). The authors concluded that in this group of patients’ hypoxic condition may have suppressed the hepcidin expression. Since hepcidin acts as a negative regulator of iron absorption and recycling it is expected that serum iron concentrations in settings of high hepcidin would be decreased, and this has been confirmed by studies (*53*, *54*).

One of the prominent laboratory features in COVID-19 is increased ferritin concentration. Hyperferritinemia in COVID-19 is a possible consequence of cytokine storm but also of increased expression of hepcidin which leads to accumulation of iron in cells. Numerous reports to date have shown that increased serum ferritin has clinical and discriminatory potential to define the severity of COVID-19 (*55*, *56*). The main question is whether ferritin is only a biological marker of uncontrolled inflammation or has a modulating role in the pathogenesis of the disease. There are some evidences that ferritin can act as a signalling molecule possibly playing an important role in inflammation. While some studies indicate immunosuppressive another suggests the possible pro-inflammatory effect of ferritin, but the immune-modulatory impact of ferritin should be confirmed by further studies (*57*, *58*). Furthermore, it has been proposed that hyperferritinemia and hepcidin up-regulation are related to cell iron toxicity by iron-dependent peroxidation and induction of ferroptosis, a distinct type of programmed cell death, which may contribute to end-organ damage in COVID-19 (*59*).

It is worth mentioning that one study found a structural similarity between a cytoplasmic tail SARS-CoV-2–spiked glycoprotein and hepcidin suggesting a possible role in changes of iron metabolism in COVID-19 (*60*). Interestingly, one study revealed that changes in iron metabolism during acute COVID-19, result in prolonged hyperferritinaemia. Increased concentrations of serum ferritin were present in 38% of patients two months after the onset of COVID-19. Hyperferritinaemia strongly correlated with serum hepcidin concentration but was also related to persisting lung pathologies in computed tomography scans and reduced physical performance on a 6-minute walking test in this group of patients (*61*).

Above mentioned studies clearly indicate a need for further investigation of iron homeostasis in COVID-19 directed to clarify the role of iron in the pathogenesis of COVID-19 and the possible benefit of the therapeutical intervention in iron metabolism.

### Interleukin-6 as a prognostic factor of severity in COVID-19 disease

Interleukin-6 is classified as a proinflammatory cytokine, mainly because it stimulates the production of acute-phase proteins in the liver and has a mild pyrogenic effect. Other effects of IL-6 are immunostimulatory. It is secreted by macrophages, monocytes, endothelial cells, T cells, and fibroblasts (*62*, *63*).

Interleukin-6 is a major mediator of the inflammatory and immune response triggered by injury or infection and its increased concentrations have been found in most patients with COVID-19 infection. It is responsible for alveolar infiltration by macrophages and monocytes, which consequently leads to respiratory failure (*64*).

Excessive activation of the immune system triggers the cytokine storm. This term represents the appearance of an aggressive, hyperimmune response to infection in which a large amount of various proinflammatory cytokines is secreted into the blood. These cytokines increase vascular permeability, cause the excretion of many cells and fluid into the pulmonary alveoli, as well as apoptosis in the pulmonary epithelium and endothelial cells (*65*).

This is the so-called immune dysregulation characterized by the activation and influx of various immune cells from the circulation to the site of infection with a destructive effect on tissue, resulting in endothelial cell destabilization, vascular barrier damage, capillary, and diffuse alveolar damage, ARDS, multiorgan failure and death. Lung injury is one of the consequences of a cytokine storm that can progress to ARDS which leads to low levels of oxygen saturation and is a major cause of death in patients with COVID-19. Although the exact mechanism of ARDS is not entirely clear, excessive production of proinflammatory cytokines is thought to be one of the main contributing factors (*66*).

Over the past few months, the role and importance of IL-6 in COVID-19 have been intensively studied. The IL-6 receptor is a target of tocilizumab, a recombinant monoclonal antibody used in the routine treatment of patients with COVID-19 (*67*).

Significant effort has been made to prove the potential role of IL-6 as a prognostic marker of mortality in patients with COVID-19 and their need for mechanical ventilation.

In one study from Spain, IL-6 was measured in 146 patients, and the aim of the study was to determine whether measuring baseline IL-6 concentration upon admission could predict the need for mechanical ventilation and response to tocilizumab treatment. The results showed that an initial IL-6 concentration > 30 pg/mL predicted the need for invasive mechanical ventilation and administration of tocilizumab (*68*). A study from Madrid showed that elevated LD, IL-6 and decreased lymphocyte values were predictive markers of mortality (*69*).

In a retrospective study from Wuhan, 1453 hospitalized patients were divided into 3 groups depending on the severity of the disease. Critical patients had significantly higher concentrations of IL-6, which confirmed the assumption that higher values correlate with disease severity (*70*).

Correlation between the initial IL-6 concentrations and development of hypoxemia during follow-up was measured in a retrospective study from Bratislava. They found the cut-off value that may be predictive for the development of hypoxemia (*71*).

In a study done in Portugal, IL-6 concentrations were measured in patients at different stages of the disease. Higher IL-6 values were found to correlate with respiratory failure and death (*72*). A similar study from Belgium showed the correlation of IL-6 in critically ill patients and death (*73*).

**Figure 1:**
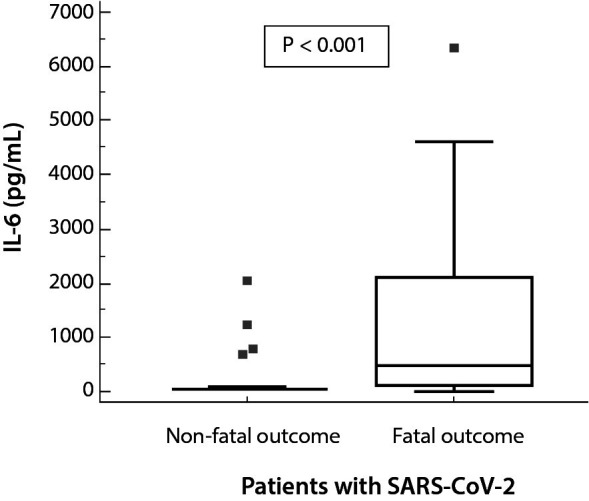
Comparison of serum IL-6 concentrations between the group with the non-fatal outcome and the group with the fatal outcome by Mann-Whitney test. The median concentration of IL-6 in the group with the non-fatal outcome was 24 pg/mL (IQR 11-53) and for the group with fatal outcome was 472 pg/mL (IQR 119-2116). IL-6 – interleukin-6. SARS-CoV-2 – severe acute respiratory syndrome Coronavirus 2. IQR – interquartile range.

In the study from University Hospital Split (unpublished data), IL-6 concentrations were measured within 3 days of hospitalization in 50 patients with acute respiratory insufficiency caused by SARS-CoV-2 infection. Out of the total number, 18 patients died, 37 patients were on mechanical ventilation during hospitalization, 12 on oxygen, and only one without assisted ventilation. Our results showed that there was a statistically significant difference in the concentration of IL-6 between non-survivors and survivors (P < 0.001) (Figure 1). There was no significant difference in IL-6 concentrations between patients on mechanical ventilation and oxygen. In the group of survivors, we observed a weak correlation between the concentration of IL-6 and days of hospitalization (P = 0.020; rho = 0.38).

Since the outbreak of this pandemic, many studies, including results from University Hospital Split, have shown that IL-6 could be used as a prognostic factor for the severity and mortality of COVID-19, and this could be helpful in the early selection of patients who are good candidates for tocilizumab/baricitinib therapy.

### Growth differentiation factor 15 in patients hospitalized for COVID 19

Growth differentiation factor 15 (GDF-15), also known as macrophage inhibitory cytokine 1 (MIC 1), is a novel cytokine that belongs to the transforming growth factor-β (TGF-β) superfamily of proteins. The members of this subfamily are named GDF 1-15. It is expressed in several tissues as the answer to oxidative stress and inflammation so in physiological conditions GDF-15 circulating concentrations are low (*74*). Mature homodimer GFF-15 is synthesized as proGDF-15, cleaved, released, and linked by disulphide bonds into circulation. However, when an inflammatory process is in progress, it was determined that GDF-15 circulating concentrations increase significantly. Growth differentiation factor 15 decreases the expression of proinflammatory cells and cytokines during tissue damage caused by inflammation, either acute or chronic (*75*). Several recent studies showed a correlation between GDF-15 and several serious illnesses, such as liver fibrosis, heart failure, stroke, and psoriasis (*76*-*78*). A detailed insight into the molecular mechanism of action has not been fully established and further researches are needed. Cytokine storm is caused by SARS-CoV-2 by binding to target cells and promotes inflammation, endothelial vascular dysfunction, and apoptosis which can lead to multiorgan failure (*75*). A relationship between GDF-15 and SARS-CoV-2 was reported recently. A Norwegian study showed that higher concentrations of GDF-15 were associated with SARS-CoV-2 viremia, hypoxemia, and worse outcomes (*79*). A Chinese study found that GDF-15 concentrations correlated with the severity of COVID-19 and the changes in its concentrations were closely associated with the disease progression so it might serve as a new biomarker for disease severity. Moreover, the authors found that expression in the convalescent group returned to concentrations comparable to healthy subjects (*79*). A study by Myhre *et al.* found that GDF-15 concentrations are not only independently associated with the risk of developing a more severe form of the disease but are also included in the pathophysiology of COVID-19 disease (*80*).

The results of these studies imply that GDF-15 concentrations could be used as a prognostic tool for outcomes of SARS-CoV-2 infection, where higher concentrations of GDF-15 would indicate a poor outcome of a disease. As mentioned, SARS-CoV-19 causes endothelial vascular dysfunction through cytokine storm, while studies showed that GDF-15 has the ability to modulate vascular contraction in endothelial damage, all of which suggests GDF-15 has another significant role in COVID-19 (*81*). Moreover, GDF-15 could have a protective value in COVID-19 disease by further investigating its role in the pathophysiology of the disease and possible therapy development.

**Figure 2:**
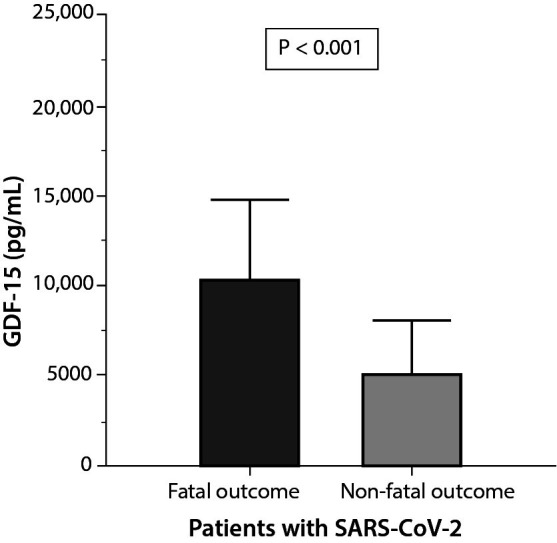
Comparison of serum GDF-15 concentrations between the group with the fatal outcome and the group with the non-fatal outcome by Student´s t-test. The mean **±** SD concentration of GDF-15 in the group with the fatal outcome (N = 35) was 10,259 **±** 4538 pg/mL and for the group with non-fatal outcome (N = 44) was 5067 **±** 2980 pg/mL. GDF-15 – Growth differentiation factor 15. SARS-CoV-2 – severe acute respiratory syndrome Coronavirus 2. SD – standard deviation.

In the University hospital Split study, GDF-15 concentrations were measured in 79 patients, 42 males, and 37 females. Median age of these patients was 78 years (range 70-82). Those patients were admitted to the hospital with acute respiratory insufficiency caused by SARS-CoV 2 infection (unpublished data). Symptoms, clinical characteristics, and medical history were obtained from the electronic medical record of patients from the BIS information system. The endpoint of the study was admission to the intensive care unit at least for 24 hours and in-hospital mortality. With the Cobas 8000 assay kit (Roche Diagnostics, Mannheim, Germany) GDF-15 concentrations were analysed. There was a statistically significant difference in serum GDF-15 concentrations depending on the clinical outcome as the group with a fatal outcome had significantly higher concentrations of serum GDF-15 compared to the group with a non-fatal outcome (P < 0.001) (Figure 2). Multivariable logistic regression showed that serum GDF-15 concentrations (OR 1.0001, 95% confidence interval (CI) 1.0000-1.0002, P = 0.046) were a significant predictor of a fatal outcome when enumerated along with baseline characteristics. The results of this study may support previous data regarding the possibility that serum GDF-15 concentrations could be used as a predictor of the outcome in patients with a severe clinical presentation of the SARS-CoV-2 infection.

This study has some limitations. First, the study group is small with narrow age range (> 65 years). Second, the study is cross-sectional, so longitudinal follow-up is missing. Finally, some other factors could influence COVID-19 patients. However, a study with a larger sample size, and a control study group is needed to completely clarify the possible influence of serum GDF-15 concentrations on outcome COVID-19 patients.

### Awake and paralysed, a case report of Guillain-Barré caused by SARS-CoV-2

The early symptomatology descriptions of the COVID-19 pandemic focused on the clinical presentations of the patent in the acute, inpatient environment. Data has emerged about some patients who continue to experience COVID-19 related symptoms after the acute period of infection. The terms ‘long COVID,’ ‘post-COVID syndrome,’ and ‘post-acute COVID-19 syndrome’ are all used to characterize this condition. The phrase ‘long haulers’ is also used (*82*-*85*).

While the definition of ‘post-acute COVID-19 syndrome’ is changing, the term is currently described as the persistence of symptoms 3 or 4 weeks following the commencement of an acute COVID-19 infection. This syndrome is further subdivided into two periods: i) clinical and laboratory abnormalities that last 4-12 weeks after acute COVID-19; and ii) clinical and laboratory abnormalities that last 12 weeks after acute COVID-19 but are not linked to other diseases (*82*, *83*).

The aetiology of post-acute COVID-19 syndrome is unknown. However, it is thought to be caused by virus-specific pathophysiological alterations, a protracted inflammatory response to the acute infection, and post-intensive care disease complications (*84*-*86*).

Clinicians and researchers are constantly learning about post-COVID conditions. Understanding them will require multi-year investigations and studies.

Guillain-Barré syndrome is a rare autoantibody-mediated neuromuscular illness characterized by a sudden and progressive paralysis following a bacterial or viral infection. During the COVID-19 pandemic, an exponential number of GBS cases were documented, implying a pathophysiological relationship between COVID-19 and GBS (*82*-*85*). In this report, a case of a 61-year-old male who presented with progressive motor weakness after COVID-19 infection.

### Case presentation

Sixty-one-year-old male without comorbidities, previously in excellent shape, presented 11 days after overcoming a mild case of COVID-19 infection. He was admitted to the Neurology department with signs of tetraparesis and tetraplegia, where treatment with plasmapheresis was initiated and then stopped due to increased inflammatory parameters and the development of pneumonia. Treatment with immunoglobulins was continued upon arrival at the intensive care unit (ICU).

During the stay, the patient was dependent on mechanical ventilation, treated several times for bacteraemia and sepsis with broad-spectrum antibiotics. He underwent a percutaneous tracheotomy (day 7) and a percutaneous endoscopic gastrostomy (PEG) (day 10). From December 24th, the patient was in contact. Communication using facial expressions and bed-side physical therapy was started. On December 31st partly spontaneous respirations were noticed. The patient understood and communicated with his lips (deliberately forming words) with emotionally reacting and fully following the flow of the interlocutor’s speech. He frowned and raised the forehead (right side weaker), with a significantly less pronounced component of right-sided peripheral facioparesis, compared to before. The tactile sensation was satisfying. The patient began localizing the touch. Limb electromyography (EMNG) showed severe sensorimotor axonal-demyelinating polyneuropathy. On January 31st, after numerous attempts, the patient was weaned from the mechanical ventilator. On the same day, the fleximetal cannula with a cuff was replaced with a plastic cannula without the cuff. After placing the phonation extension on the cannula, the patient began to speak. His muscular strength was improving dramatically. On February 4th, decannulation was performed. A urinary catheter was removed, and the patient had an urge and urinated spontaneously. On February 8th, PEG was removed, and the patient was fed *per os* ever since. Daily improvement in motor skills was noticeable (raising both hands in the air, raising the pelvis, flexing and extending the feet, and moving the legs). The patient was in good general condition, hemodynamically stable, with declining inflammatory parameters and spontaneous diuresis. Tracheostomy and PEG wounds were normal. The motor progress of the patient was visible on a daily basis, and he was transferred to the Department of Physical Medicine in order to intensify physical therapy and training after 61 days spent in the ICU. A month after his discharge from the ICU, with help, the patient is walking on his own.

Since SARS-CoV-2 produces a high number of severe infections, patients with neurological complications may go unnoticed, just as primarily neurological patients may become infected or develop asymptomatic COVID 19 infection. Guillain-Barré syndrome is a potentially life-threatening, antibody-mediated disease of the peripheral nerves (*82*-*84*). The disease can progress quickly, with numerous patients experiencing respiratory failure that necessitates mechanical ventilation. Guillain-Barré syndrome, in the context of COVID-19, has been seen in an increasing number of case reports (*83*, *84*). The mechanism through which SARS-COV-2 develops GBS remains unknown. The SARS-COV-2 stimulates leukocytes, prompting them to release a high amount of cytokines, further triggering the inflammatory cascade and producing significant tissue damage with diverse organ dysfunction, according to ongoing research (*82*). This route is thought to explain neurologic impairment in COVID-19 aetiology (*82*-*84*).

### Coronavirus Disease 2019 impact on fertility and assisted reproduction

The SARS-CoV-2 virus affects many organs, and it has also been assumed that the virus might influence human reproductive systems. The virus attacks target host cells by binding to the ACE2 after which the transmembrane serine protease 2 (TMPRSS2) cleaves S-protein to facilitate its entry into the cell (*87*). Tissues and cells with high expression of ACE2 receptor and TMPRSS2 are more vulnerable to infection by SARS-CoV-2. Studies have shown that the ACE2 gene is expressed in the ovary, where it is supposed to regulate hormone secretion, follicle development, and oocyte maturation (*88*, *89*). A study by Wu *et al.* demonstrated co-expression of ACE2 and TMPRSS2 in human ovaries suggesting that SARS-CoV-2 infection may affect ovarian function by directly binding to the ACE2/TMPRSS2 (*90*). Furthermore, a significant number of studies have shown that inflammation affects ovarian function indirectly by the action of cytokines (*91*). In the uterus, ACE2 is expressed at a low-level making infection of this organ unlikely (*92*). There is also the possibility that SARS-CoV-2 infection might affect female fertility by direct infection of ovarian granulosa cells and/or oocytes, in that way negatively affecting ovarian function and reducing oocyte quality (*93*). Some IVF methods also include breaking the zona pellucida for fertilization (intracytoplasmic sperm injection), which represents a theoretical opportunity for the virus to gain access to embryonic cells. The study of Wang *et al.* implies that there is no impact on female fertility, embryo laboratory outcomes, or clinical outcomes in ART (assisted reproductive technology) treatments in females with a history of asymptomatic or mild SARS-CoV-2 infection (*94*). But the effect of severe disease on female fertility is not explored yet.

The SARS‐CoV‐2 infection can possibly affect male fertility by direct virus-induced damage of the testis, epididymis, and spermatogonia (*95*). According to the studies ACE2 has a role in the regulation of steroidogenesis and spermatogenesis in male testicles (*96*). Spermatogonia, Leydig cells, and Sertoli cells have been found to contain ACE2 rendering them potential SARS-CoV-2 targets (*97*). A previous study has shown that testicular damage, orchitis, and sterility are possible complications of SARS‐CoV which share the same binding receptor as SARS‐CoV‐2 and have 80% sequencing similarity (*98*). Furthermore, there is also the possibility of indirect damage to male fertility during SARS‐CoV‐2 infection mediated by inflammation/cytokines, oxidative stress, antibodies, and high fever (*97*). Long term influence of these well-known factors that impair male fertility in patients with a history of SARS-CoV-2 infection has not yet been examined.

The COVID-19 pandemic affects all medical specialties, including the field of reproductive medicine. Many clinical questions regarding the influence on reproduction remain unanswered yet. Results of some studies indicate the possible influence of SARS-CoV-2 infection on human reproduction, so short and long-term effects of infection, severe ones, and especially mild forms of the disease which affect a huge number of patients need to be further investigated. If future studies confirm the influence of SARS-CoV-2 infection on human reproductive organs and fertility the term of the long COVID could assume a whole new meaning.
